# Human thyroid cancer cells as a source of iso-genic, iso-phenotypic cell lines with or without functional p53

**DOI:** 10.1038/sj.bjc.6690177

**Published:** 1999-03

**Authors:** F S Wyllie, M F Haughton, J M Rowson, D Wynford-Thomas

**Affiliations:** Cancer Research Campaign Laboratories, Department of Pathology, University of Wales College of Medicine, Heath Park, Cardiff CF4 4XN, UK

**Keywords:** p53, differentiation, iso-genic, tumour progression, thyroid

## Abstract

Differentiated thyroid carcinomas (in contrast to the rarer anaplastic form) are unusual among human cancers in displaying a remarkably low frequency of p53 mutation and appear to retain wild-type (wt) p53 function as assessed by the response of derived cell lines to DNA damage. Using one such cell line, K1, we have tested the effect of experimental abrogation of p53 function by generating matched sub-clones stably expressing either a neo control gene, a dominant-negative mutant p53 (143^ala^) or human papilloma virus protein HPV16 E6. Loss of p53 function in the latter two groups was confirmed by abolition of p53-dependent ‘stress’ responses including induction of the cyclin/CDK inhibitor p21WAF1 and G1/S arrest following DNA-damage. In contrast, no change was detected in the phenotype of ‘unstressed’ clones, with respect to any of the following parameters: proliferation rate in monolayer, serum-dependence for proliferation or survival, tumorigenicity, cellular morphology, or tissue-specific differentiation markers. The K1 line therefore represents a ‘neutral’ background with respect to p53 function, permitting the derivation of functionally p53 + or − clones which are not only iso-genic but also iso-phenotypic. Such a panel should be an ideal tool with which to test the p53-dependence of cellular stress responses, particularly the sensitivity to potential therapeutic agents, free from the confounding additional phenotypic differences which usually accompany loss of p53 function. The results also further support the hypothesis that p53 mutation alone is not sufficient to drive progression of thyroid cancer to the aggressive anaplastic form. © 1999 Cancer Research Campaign


*British Journal of Cancer* (1999) **79**(7/8), 1111–1120
© 1999 Cancer Research Campaign
Article no. bjoc.1998.0177

### Human thyroid cancer cells as a source of iso-genic, iso-phenotypic cell lines with or without functional p53

**FS Wyllie, MF Haughton, JM Rowson and D Wynford-Thomas**

Cancer Research Campaign Laboratories, Department of Pathology, University of Wales College of Medicine, Heath Park, Cardiff CF4 4XN, UK

**Summary** Differentiated thyroid carcinomas (in contrast to the rarer anaplastic form) are unusual among human cancers in displaying a remarkably low frequency of p53 mutation and appear to retain wild-type (wt) p53 function as assessed by the response of derived cell lines to DNA damage. Using one such cell line, K1, we have tested the effect of experimental abrogation of p53 function by generating matched sub-clones stably expressing either a neo control gene, a dominant-negative mutant p53 ($143^{ala}$) or human papilloma virus protein HPV16 E6. Loss of p53 function in the latter two groups was confirmed by abolition of p53-dependent 'stress' responses including induction of the cyclin/CDK inhibitor p21WAF1 and G1/S arrest following DNA-damage. In contrast, no change was detected in the phenotype of 'unstressed' clones, with respect to any of the following parameters: proliferation rate in monolayer, serum-dependence for proliferation or survival, tumorigenicity, cellular morphology, or tissue-specific differentiation markers. The K1 line therefore represents a 'neutral' background with respect to p53 function, permitting the derivation of functionally p53 + or – clones which are not only iso-genic but also iso-phenotypic. Such a panel should be an ideal tool with which to test the p53-dependence of cellular stress responses, particularly the sensitivity to potential therapeutic agents, free from the confounding additional phenotypic differences which usually accompany loss of p53 function. The results also further support the hypothesis that p53 mutation alone is not sufficient to drive progression of thyroid cancer to the aggressive anaplastic form.

**Keywords:** p53; differentiation; iso-genic; tumour progression; thyroid

Mutation of the tumour suppressor gene p53 leading to loss of function (with or without dominant-negative activity), is well-established as a key event in human tumorigenesis (Greenblatt et al, 1994). The high frequency of mutation observed in most cancers clearly reflects a strong selective advantage, which is supported by both in vitro (Michalovitz et al, 1991) and in vivo transgenic experiments (Lavigeur et al, 1989; Kemp et al, 1993). Indeed, many cancer cells appear to be totally dependent on a loss of p53 function for survival (Baker et al, 1990; Diller et al, 1990; Johnson et al, 1991).

It is clear, however, that with a few possible exceptions such as ultraviolet (UV)-induced skin cancer (Zeigler et al, 1994), p53 mutation is not an initiating event and only confers a selective growth advantage once a critical stage in the clonal evolution of a tumour has been reached (Fearon and Vogelstein, 1990; Kemp et al, 1993). This correlates loosely with increasing clinical 'stage' and pathological 'grade' (Fujimoto et al, 1992; Barnes et al, 1993; Navone et al, 1993), but the exact nature of the step that converts a p53-insensitive to a p53-sensitive tumour clone remains unclear, as indeed does the biochemical basis for selective growth suppression of such tumour cells by wild-type (wt) p53 (Wynford-Thomas, 1996; Wynford-Thomas et al, 1996).

Tumours of the thyroid follicular cell provide a particularly useful model for studying this question. Instead of the usual continuous spectrum, in these tumours there is a remarkably clear-cut demarcation between two clinico-pathological entities: i) differentiated cancers (the majority) which usually carry a very favourable prognosis; and ii) undifferentiated (anaplastic) cancers which are among the most malignant of all human cancers (Carcangiu et al, 1985; Williams and Williams, 1989). Furthermore, the rate of transition between these phenotypes appears to be very low, such that most differentiated cancers can metastasize and (in the absence of therapy) generate a potentially fatal tumour burden without ever undergoing anaplastic transformation. This 'discontinuity' in progression correlates with an equally striking difference in frequency of p53 mutation, from virtually zero in differentiated cancers (whether primary or metastatic) to well over 50% in anaplastic tumours (Ito et al, 1992; Fagin et al, 1993; Wynford-Thomas, 1993). The magnitude of this contrast, coupled with the stability of the differentiated stage, enabling homogeneous clinical samples, and even cell lines, with wt p53 phenotype to be obtained, makes this a unique model to investigate the nature of the selection pressure driving p53 mutation in human cancer.

The correlation between p53 mutation and the anaplastic phenotype could most readily be explained if loss of p53 function led directly to the increased tumour aggressiveness and loss of differentiation characteristic of this progression step. Evidence from our laboratory, however, indicates that this simple scenario is not correct. Using a cell line derived from a differentiated thyroid cancer (K1), in which wt p53 appeared to be functional in terms of the response to 'acute' stimuli from DNA-damaging agents such as bleomycin (Wyllie et al, 1995), we previously reported that stable expression of a dominant-negative mutant of p53 ($143^{ala}$), while abrogating the G1/S cell cycle check-point, had no other obvious phenotypic effect (Blaydes et al, 1995; Wyllie et al, 1995).

This is a potentially important finding which deserves further investigation, since it supports the hypothesis that progression

*Received 27 April 1998*
*Revised 8 July 1998*
*Accepted 13 July 1998*

*Correspondence to:* D Wynford-Thomas from differentiated to anaplastic thyroid cancer requires events additional to loss of p53 function. Furthermore, we reasoned that if K1 cells were indeed indifferent to the presence or absence of wt p53 function, they would represent an ideal, 'neutral', background on which to derive matched functionally p53 + or – lines for use as a tool to investigate p53-dependency of therapeutic agents.

Our earlier study (Wyllie et al, 1995) examined only a few phenotypic features, however, and employed just a single p53 mutant which has subsequently been found to be an inefficient dominant-negative in some contexts (Williams et al, 1995). We have therefore now re-examined this model in more detail, analysing multiple clones, multiple parameters of proliferation and differentiation state, and using not only mutant p53 but also the human papilloma virus protein HPV16 E6 as a means of abrogating p53 function.

### MATERIALS AND METHODS

### Cell culture

The K1 and FTC human thyroid cancer cell lines were kindly provided by Prof. M Schlumberger (Villejuif, France) and Dr P Goretski (Dusseldorf, Germany), respectively. Both lines and their derivatives were grown as monolayers in a 2:1:1 (by volume) mixture of Dulbecco's modified Eagle's medium, Ham's F12, and MCDB104 (all from Life Technologies, Paisley, UK) (Bond et al, 1992), supplemented with 10% fetal calf serum (FCS; Imperial Labs, London, UK).

### DNA transfection

Cells were plated at $2 \times 10^5$ per 60-mm dish and transfected 2 days later by the strontium phosphate coprecipitation method (Brash et al, 1987). The plasmids used were pc53-SCX3 which expresses a human mutant p53 ($143^{ala}$) (Baker et al, 1990) and pSV2neo as a negative control (Southern and Berg, 1982). Stable transfectants were selected by growth in 400 μg $ml^{-1}$ G418 (Life Technologies).

### Retroviral gene transfer

A high-titre amphotropic retroviral vector (PA317-16E6) (Halbert et al, 1991), expressing the E6 gene from human papilloma virus type 16, was kindly provided by Dr D Galloway (Seattle, WA, USA). As a negative control we used a neo-only vector of similar titre, psi-CRIPneo (Wyllie et al, 1993).

For gene transfer, cells were plated at $2 \times 10^5$ per 60-mm dish and infected with undiluted viral supernatant (containing 8 μg $ml^{-1}$ polybrene, Aldrich, Gillingham, UK) from amphotropic producer cell lines as described previously (Burns et al, 1989). Two days later, cultures were passaged into G418 (400 μg $ml^{-1}$) and colonies subsequently selected.

### Immunocytochemical analysis

For p53 detection, cells growing on thermanox coverslips (Life Technologies) were fixed in methanol:acetone (1:1) (10 min at –20°C) and immunostained with the mouse monoclonal antibody PAb421 (Harlow et al, 1981) using a standard indirect immunoperoxidase procedure (Wynford-Thomas et al, 1990). For p21WAF1 detection, coverslips were fixed in 4% paraformaldehyde (10 min), then pretreated with 100 mM glycine (10 min), 0.2% Triton X-100 (20 min) and 0.3% hydrogen peroxide (3 min) and non-specific binding blocked with 2% horse serum (30 min). p21 protein was detected using mouse monoclonal antibody 6B6H4 (Pharmingen Inc, San Diego) followed by a mouse-specific avidin–biotin–peroxidase system (Novocastra, Newcastle-upon-Tyne, UK).

### ELISA

Cellular p53 protein content was determined by enzyme-linked immunosorbent assay (ELISA) using a 'pan-p53' kit (Oncogene Science, New York, NY, USA) as described previously (Wyllie et al, 1995).

### DNA damage

Bleomycin (Lundbeck, Milton Keynes, UK) was prepared as a 7.5 mg $ml^{-1}$ stock solution in de-ionized water, stored at –20°C and used at a final concentration of 250 μg $ml^{-1}$, which was previously determined as the minimal dose required to obtain maximum growth arrest in these cells (Wyllie et al, 1995). The timing of analysis of p21WAF1 expression following bleomycin treatment was based on previous time-course studies (Bond et al, 1995).

### Flow cytofluorimetry

This was performed as described previously (Wyllie et al, 1995).

### Northern blot analysis

Total RNA was extracted from cells in log-phase growth by a modified guanidium/phenol method (RNAzol B; AMS Biotechnology, Witney, UK), separated on 1% agarose gels (10 μg per lane) and blotted to Hybond $N^+$ (Amersham, Aylesbury, UK). As a positive control for thyroid-specific transcripts, RNA was similarly extracted from thyroid tissue derived from a patient with Grave's disease. The following DNA probes were used: for PAX-8, a 0.3 kb HindIII/EcoR1 insert of the human cDNA clone, H26P/S3 (Poleev et al, 1992); for TTF1, a 0.7 kb Sac1 insert of the rat cDNA clone, TTF1-THA (Guazzi et al, 1990); for thyroglobulin (TG), a 0.98 kb Pst1 insert of the human cDNA clone, phTg1 (Brocas et al, 1982); for thyroid peroxidase (TPO), a 3 kb EcoR1 insert of the human cDNA clone, pTPO (Libert et al, 1987); for the thyrotropin receptor (TSHR), a 2.3 kb BamH1/Xbo1 insert of the human clone pSVL (Libert et al, 1989) and for GAPDH (control), a 1.2 kb Pst1/Xba1 insert of the human cDNA clone, pHcGAP (ATCC, Rockville, MD, USA). Filters were washed at high stringency (65°C; 0.1 × SSPE) for all probes except for TTF1, which was washed at low stringency (65°C; 1 × SSPE), and were stripped and rehybridized to the GAPDH probe as a 'loading' control.

### Analysis of cell growth

#### *Doubling time*

Cells ($3 \times 10^4$) were plated in 60-mm dishes in medium containing 10% FCS, cell number counted daily until the cultures reached confluence and doubling time calculated during log-phase growth.

#### *Proliferation assay*

Cells undergoing DNA synthesis were identified by addition of the thymidine analogue bromo-deoxyuridine (BrdU) (Dako, Glostrup,

 © Cancer Research Campaign 1999

Denmark) to the medium for 1 h at a final concentration of 10 μM. After labelling, cells were fixed in 70% ethanol (30 min at 4°C), then pretreated with 4 M HCl (10 min) and 0.1 M borax, pH 8.5 (5 min). Incorporated BrdU was detected by an immunoperoxidase method incubating first for 60 min with a mouse anti-BrdU primary antibody (Dako) in the presence of 25 U $ml^{-1}$ DNase1 (Life Technologies), followed by peroxidase labelled rabbit anti-mouse immunoglobulin (Dako) and finally diaminobenzidine substrate. After counterstaining with haematoxylin, the proportion of positive nuclei (LI) was determined, using a sample of > 1000 cells per data point.

*Colony forming efficiency*

A single cell suspension was obtained after trypsinization and $10^2$ cells were added to duplicate 60-mm dishes. After 10 days in standard growth conditions Giemsa-stained colonies of 50 cells or more were counted, and colony forming efficiency (CFE) expressed as a % of cell number plated.

### Analysis of cell death

*Terminal deoxynucleotidyl transferase assay*

Cells were seeded on coverslips and fixed with 4% paraformaldehyde (30 min). Endogenous peroxidase was blocked with 0.3% hydrogen peroxide in methanol (30 min) and the cells permeabilized with 0.1% Triton X-100 in 0.1% sodium citrate (15 min). Cells were then incubated for 1 h at 37°C with 250 U $ml^{-1}$ terminal deoxynucleotidyl transferase (TdT; Promega, Southampton, UK) and 1 nmole biotin–16–dUTP (Boehringer Mannheim, Lewes, UK). Sites of biotin–16–dUTP localization were visualized using the mouse specific avidin–biotin–peroxidase system (Novocastra). Cells were counterstained with haematoxylin and the proportion of apoptotic (brown) cells assessed, in samples of > 1000 cells per data point.

*Conventional gel electrophoresis*

DNA was extracted according to Wyllie et al (1989) from a combination of free-floating and loosely-attached cells, electrophoresed in a 1.5% agarose gel for 1–2 h at 90 V and visualized by ethidium bromide staining.

### Tumorigenicity

$10^6$ cells, suspended in 0.2 ml of growth medium were injected subcutaneously into athymic (nude) mice (1 injection per animal) and monitored for appearance of tumours for up to 8 months.

### RESULTS

### Derivation of sub-clones of the thyroid cancer line K1 expressing mp53

The K1 cell line (Wyllie et al, 1993; Challeton et al, 1997) was derived by spontaneous immortalization from the most common pathological sub-type of differentiated thyroid cancer – 'papillary' carcinoma (Williams and Williams, 1989). Conventional immunocytochemical (ICC) analysis with PAb421 showed low levels of nuclear p53 and sequencing of the entire coding region (exons 2–11) from reverse transcribed mRNA confirmed the presence of only wt sequence (Wyllie et al, 1995).

To directly assess the effect of loss of wt p53 function on the behaviour of K1 cells, sub-clones (designated K1scx) were derived by stable transfection with plasmid pc53-SCX which express the $ala^{143}$ mutant of human p53. Control sister clones (K1neo) were obtained using plasmid pSV2neo. Four K1scx clones (K1scx3, 6, 8 and 9) were initially chosen on the basis of their much higher expression of total p53 protein as shown by immunocytochemistry (Figure 1B), when compared to the four K1neo control clones (3, 4, 5 and 11) (Figure 1A). (Mutant p53 is known to be stabilized in these cells.) This was confirmed by ELISA assay which showed p53 protein content in clones scx3, 6 and 8 to be 175, 222 and 164 μg $mg^{-1}$ total cellular protein, respectively, whereas in K1neo clones it could not be reliably detected above the detection limit (approximately 5 μg $mg^{-1}$). For comparison, FTC133, a transformed thyroid cell line known to contain a homozygous mutant p53 gene (Wright et al, 1991), contained 80 μg p53 per mg total protein.

### Functional status of p53 in K1scx subclones p53 function in K1scx clones was assessed by determining the integrity of p53-dependent DNA damage responses.

A B

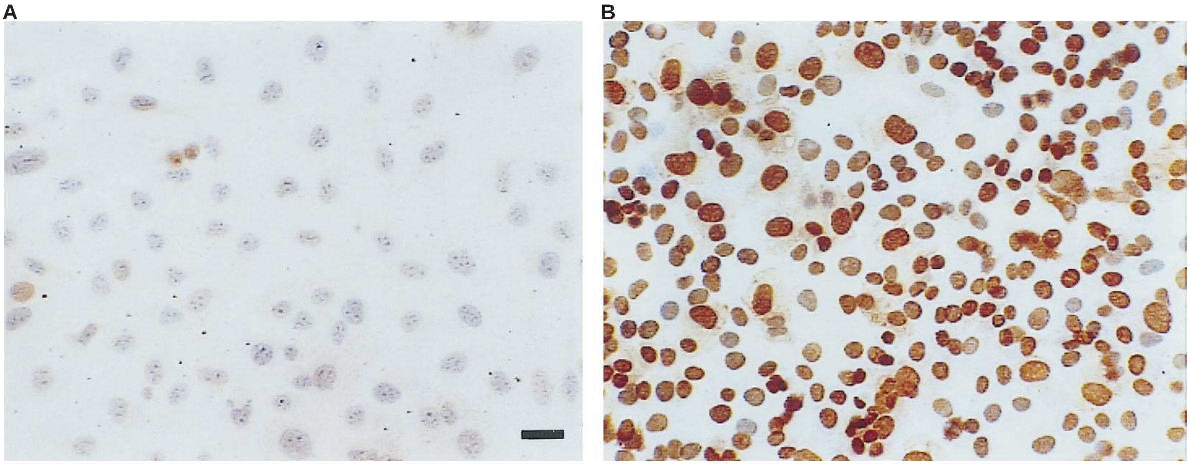

**Figure 1** Immunocytochemical analysis using antibody PAb421 showing high expression of p53 protein in nearly all cells of a representative sub-clone of K1 cells (K1scx6) derived by transfection of ala143 mutant p53 (**B**), compared to low expression in control sub-clone K1neo3 (**A**). Immunoperoxidase; haematoxylin counterstain; bar = 50 μm

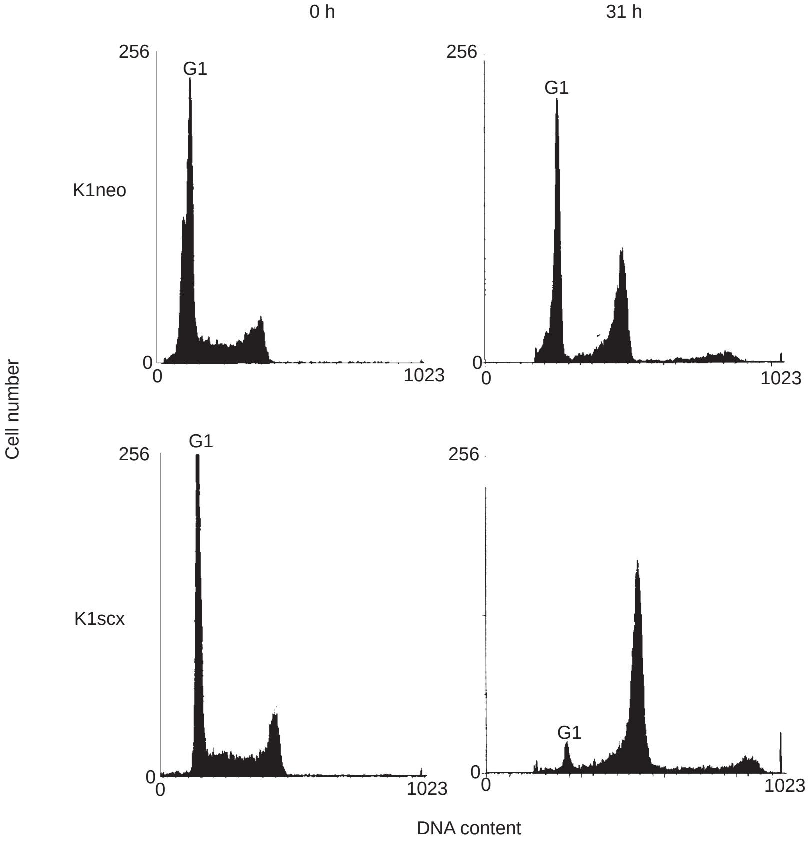

**Figure 2** Flow cytometric analysis demonstrating absence of normal G1 arrest in K1scx cells following DNA damage. Note loss of the G1 DNA peak 31 h after bleomycin treatment in K1scx6 compared to control clone K1neo3

p53 has been identified as a critical component of a signalling pathway leading to G1 arrest following exposure to ionizing radiation (Kastan et al, 1991). The status of this checkpoint was therefore assessed in K1neo and K1scx clones by analysing the proportions of cells in G1, S and G2/M cell cycle phases following treatment with the radiomimetic agent, bleomycin (Hsu et al, 1989; Wyllie et al, 1995).

In K1neo clones, flow cytometry showed no significant reduction in the G1 fraction up to 31 h after bleomycin treatment, by which time most of the cells that were initially in S phase had progressed into, and remained in, the G2 fraction (Figure 2). This is consistent with retention of both G1 and G2 cell cycle checkpoints. In contrast, all four K1scx clones showed a major reduction of the G1 fraction by 31 h after bleomycin treatment, with most of the cells having moved into the G2 compartment (Figure 2), indicating retention of the G2/M, but loss of the G1/S checkpoint.

One of the downstream transcriptional targets of p53 responsible for this G1 arrest is the cyclin kinase inhibitor, p21WAF1 and cells that have lost functional p53 fail to induce expression of p21WAF1 in response to some forms of DNA damage (El-Deiry et al, 1994). Intranuclear p21WAF1 protein content in K1neo and K1scx clones was therefore assessed immunocytochemically before and after bleomycin treatment.

Untreated K1neo clones showed marked cell–cell heterogeneity with an average 4.5 ± 0.9% of nuclei (mean ± SE for the four clones tested) showing detectable immunostaining (Figure 3A). Bleomycin treatment led to a clear increase in expression, in terms both of the proportion of positive nuclei (which increased to 21.9 ± 1.7%) and the intensity of immunostaining (Figure 3B and Table 1). Untreated K1scx cells showed a very similar level of immunostaining to K1neo cells (average of 4.4 ± 1.5% for the four clones; Figure 3C). In contrast, however, in K1scx cells (all four clones), no induction of expression could be detected in treated cultures either in proportion positive (3.4 ± 0.4% for the four clones) or intensity (Figure 3D and Table 1).

### Derivation of K1 lines expressing HPVE6

Although the above data supported the effectiveness of our ala143 mutant as a dominant-negative, there remained some concern from other studies (Forrester et al, 1995; Friedlander et al, 1996; Ludwig et al, 1996) that some p53 targets may still be activatable in K1scx cells, due either to their greater sensitivity to any residual wt p53 function, and/or to retention of transcriptional activity by the mutant for a sub-set of p53-dependent promoters.

We therefore also used an independent approach for abrogation of wt p53 function in K1 cells, by expression of the HPV16E6 gene introduced by means of an amphotropic retroviral vector, pLXSNE6 (Halbert er al, 1991). Five G418 resistant clones were chosen at random together with four control lines expressing only a retrovirally-transduced neo gene and p53 functional status was assessed as above by measuring p21WAF1 induction in response to bleomycin treatment.

The neo controls behaved, as expected, like their transfected counterparts, the proportion of p21-positive nuclei rising from 5.0 ± 2.0% to 25.1 ± 5.1% following bleomycin treatment (data

 *© Cancer Research Campaign 1999*

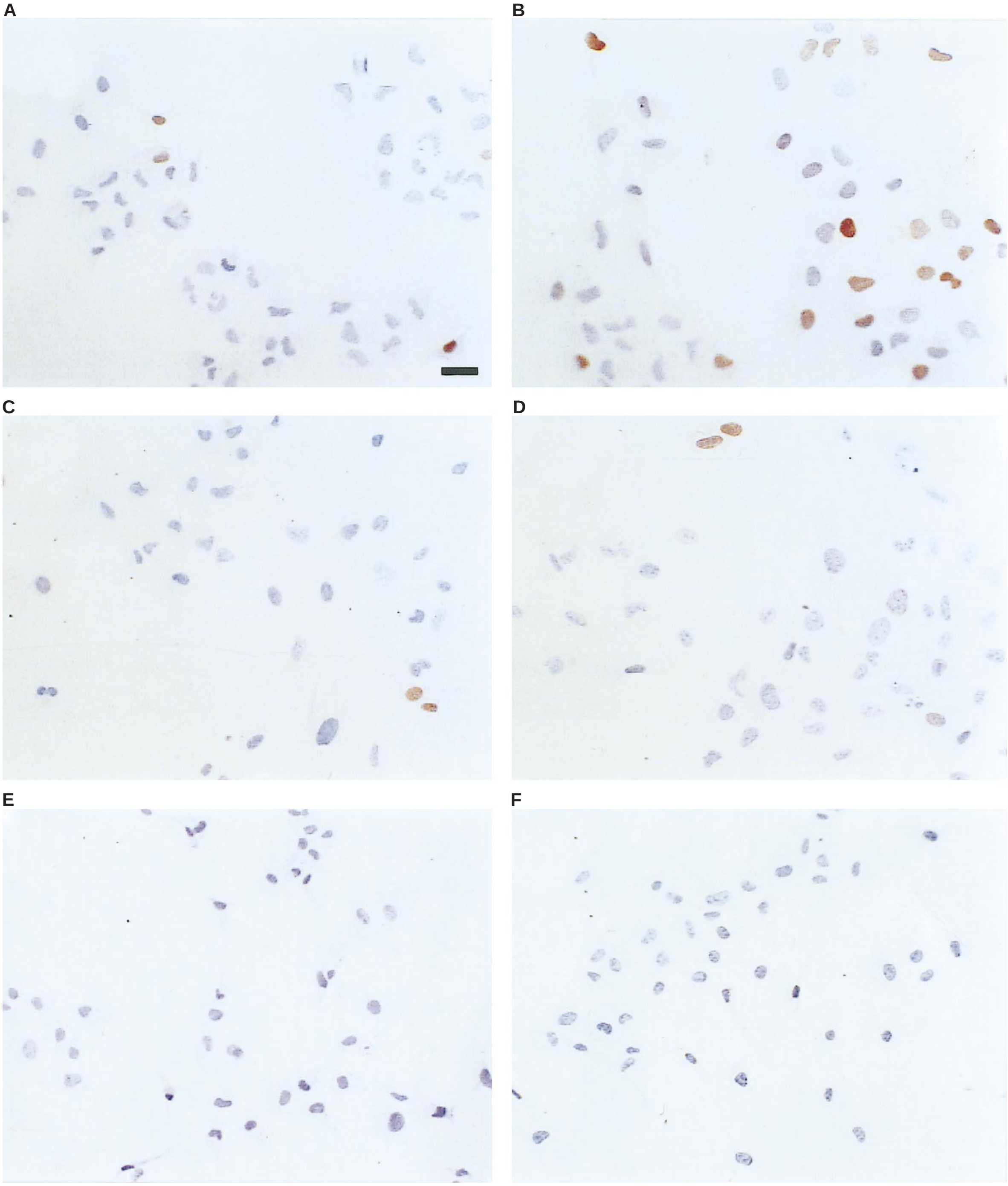

**Figure 3** Abrogation by mutant p53 or HPV16E6, of p21WAF1 induction in K1 cells following exposure to bleomycin. Immunocytochemical analysis of p21 expression in control clone K1neo3 (**A, B**) compared to K1scx6 (**C, D**) and K1E6-4 (**E, F**), in untreated cultures (**A, C, E**) and 4 h after the start of bleomycin treatment (**B, D, F**). Haematoxylin counterstain; bar = 50 μm not shown). However, a clear difference was observed between K1.E6 clones and their K1scx counterparts, in that in untreated cultures *no* p21-expressing cells could be detected in three out of five K1.E6 clones and only a very low % in the other two clones (the average for all five clones being < 0.1%; Figure 3E). This is consistent with a more complete abrogation of p53 function by E6 than by the $143^{ala}$ p53 mutant. As expected, E6 clones showed no induction of p21 expression following bleomycin treatment (Figure 3F and Table 1).

### Effect of inactivating wt p53 function on growth and survival of K1 cells

The doubling time (mean ± SE) in standard medium during log growth was 22.0 ± 1.8 h for the three K1neo clones and 24.3 ± 0.8 h for the three K1scx clones analysed (Table 2). The proportion of nuclei in S phase (LI) under the same conditions was 34.0 ± 0.75% and 34.6 ± 1.0%, respectively (Table 2). All clones showed a very high plating efficiency, CFE being 60.0 ± 16.2% for K1neo

**Table 1** Loss of wt p53 function in K1 clones expressing mp53 or HPV16E6 as evidenced by failure to induce p21WAF1 protein expression following exposure to bleomycin

| | % of cells with immunodetectable p21 | |
|---|---|---|
| **Clone** | **– bleomycin** | **+ bleomycin** |
| Control | | |
| neo3 | 2.7 | 21.4 |
| neo4 | 5.8 | 24.2 |
| neo5 | 6.1 | 24.6 |
| neo11 | 3.3 | 17.2 |
| mp53 | | |
| scx3 | 8.5 | 4.6 |
| scx6 | 1.8 | 3.0 |
| scx8 | 2.7 | 2.8 |
| scx9 | 4.5 | 3.0 |
| HPV E6 | | |
| E6.1 | 0 | 0 |
| E6.2 | 0 | 0 |
| E6.3 | 0.2 | 0.2 |
| E6.4 | 0 | 1.2 |
| E6.5 | 0 | 0 |

compared with 70.5 ± 14.3% for K1scx clones (Table 2). None of these differences between K1neo and K1scx reached statistical significance.

The effect of inactivation of wt p53 function on response to withdrawal of serum growth factors was tested by plating cells in medium supplemented with 10% serum for 48 h and then refeeding with serum-free media. Assessment of DNA synthesis was performed only after 2 days serum starvation since later analysis proved unreliable due to high cell death rates. Most clones showed only a small decrease in LI, apart from K1neo3 which showed an approximately threefold decrease (Table 2). Overall, there was no significant difference in LI between the four K1neo and the four K1scx clones in serum-starved conditions (23.2 ± 5.0% and 29.4 ± 1.75%, respectively) (Table 2).

Determination of cell number at 0, 2, 5, 7 and 9 days after serum starvation showed an increase in most neo and scx clones up to day 5 and a decrease thereafter. There was considerable inter-clonal variability in the rate of onset of this decrease in cell number with a trend for the K1scx clones to lose cells more quickly than the K1neo clones (Figure 4). However, this difference did not reach statistical significance. The basis for this cell loss was investigated in one K1neo clone (No. 3) and one scx clone (No. 6). The TdT assay showed an increase in the number of positive cells from < 1% at days 0 or 2, to 2.5–3.0% at day 5 and 6.5–14% by day 7 and 9 of serum-starvation, consistent with apoptosis being the mode of cell death. This was confirmed by gel electrophoretic analysis of DNA extracted from floating and loosely attached cells obtained from a pool of day 7 and 9 serum-starved cultures, which showed the laddering pattern characteristic of apoptosis (data not shown).

### *Tumorigenicity*

All clones formed visible tumours in athymic mice within 7 months. The median time taken to form 0.5 cm diameter tumours was 4 weeks in K1scx compared to 5 weeks for K1neo. This difference was entirely attributable to a long latency in two of the animals injected with clone neo3 (Table 2) and, overall, did not reach statistical significance.

**Table 2** Growth parameters for K1neo and K1scx clones

| Clone | Doubling time (hours) | BrdU LI (%) | | CFE (%) | Tumour latency (weeks)[a] |
|---|---|---|---|---|---|
| | | 10% FCS | 0% FCS | | |
| neo3 | 21.7 | 32.6 | 10.3 | 48 | 5, 7, 20, 28 |
| neo4 | 19.1 | 34.0 | 29.9 | 40 | 4, 4, 5, 5 |
| neo5 | 25.2 | 36.0 | 20.7 | 92 | 5, 5, 6, 8 |
| neo11 | N.D. | 33.3 | 31.9 | N.D. | ND |
| scx3 | 23.0 | 32.7 | 26.3 | 30 | ND |
| scx6 | 24.1 | 34.7 | 26.8 | 75 | 4, 4, 5, 8 |
| scx8 | 25.8 | 33.6 | 30.8 | 80 | 2, 3, 4, 4 |
| scx9 | N.D. | 37.2 | 33.7 | 97 | ND |

ND, Not determined. [a]Time to form tumour with maximum diameter 0.5 cm.

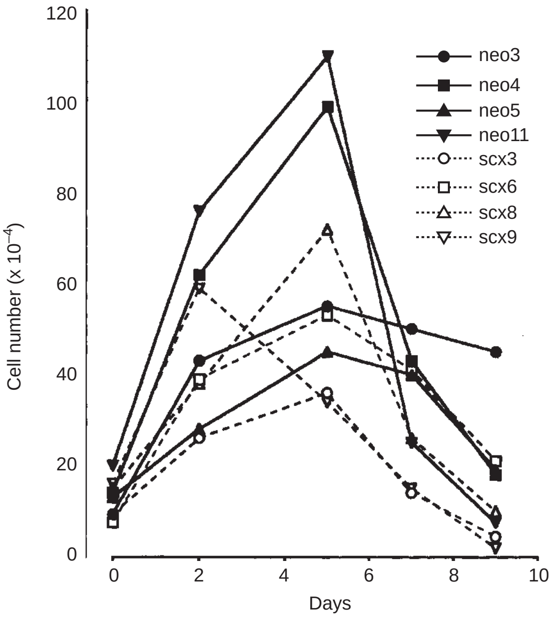

**Figure 4** Effect of serum-starvation on growth and survival of control and mp53-expressing K1 sub-clones. Cell number is shown for four K1neo clones (solid lines) and four K1scx clones (broken lines) at 0, 2, 5, 7 and 9 days after serum withdrawal

### Effect of loss of functional p53 on differentiation in K1 cells

### *Morphology*

Under standard growth conditions all K1neo clones grew as a monolayer with well defined cell–cell boundaries and although at least some cells still continued to cycle at confluence (as indicated by the 24-h BrdU LI; data not shown), this did not result in any appreciable ‘piling up’ (see Figure 5A for a representative neo clone). The expression of mutant p53 or HPVE6 did not result in any consistent change in morphology (see Figure 5b for a representative scx clone).

### *Expression of thyroid-specific differentiation*

Previous studies have suggested that altering p53 functional status can lead to an alteration in differentiation, as measured by the expression of the thyroid-specific transcription factor, PAX8

*© Cancer Research Campaign 1999*

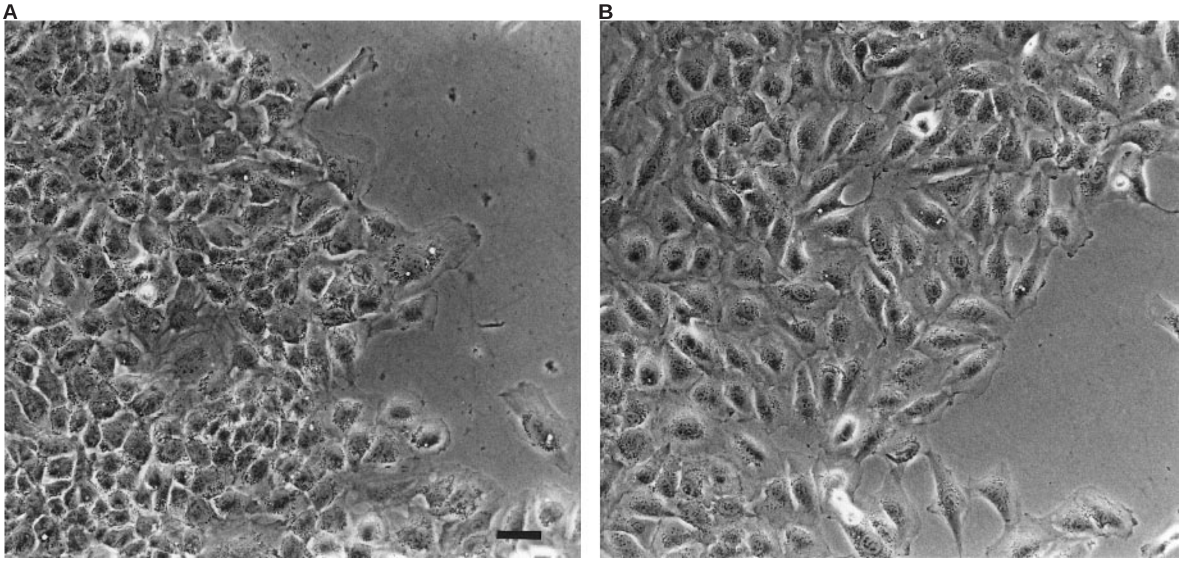

**Figure 5** Abrogation of p53 function does not cause morphological change in K1 cells. Phase contrast photomicrogaphs of representative control clone K1neo3 (**A**) and mp53-expressing clone K1scx6 (**B**); bar = 50 µm

(Battista et al, 1995; Fagin et al, 1996). To investigate this relationship in our cell line, the K1scx, K1E6 and K1neo clones were compared and the ratio of PAX8 to control (GAPDH) mRNA expression was calculated for each clone. No significant difference in PAX8 mRNA expression between the four K1neo and the four K1scx clones was observed (mean ratio of 1.0 ± 0.4 and 0.9 ± 0.3, respectively; Figure 6A). A similar result was seen when the five K1E6 clones were compared with their four control neo clones (mean ratio of 1.6 ± 0.7 for E6 and 1.7 ± 0.8 for neo; Figure 6B). For comparison, a poorly differentiated thyroid carcinoma cell line (FTC133), showed no PAX8 expression (Figure 6A and B).

### DISCUSSION

In this study we have used gene transfer in vitro to directly investigate whether loss of wt p53 function is sufficient to drive progression of the transformed phenotype in thyroid cancer cells. Expression of the $143^{ala}$ mutant failed to cause any demonstrable loss of differentiation in a cell line derived from differentiated thyroid (papillary) cancer, nor did it confer any evidence for increased proliferative capacity in vitro or in vivo.

There is now good evidence that expression of such mutants can sometimes fail to eliminate function of the endogenous wt protein and, indeed, the effectiveness of their dominant-negative activity appears to be highly dependent on cell context, mutation site (Forrester et al, 1995; Friedlander et al, 1996; Ludwig et al, 1996), and promoter target. In our study, the $143^{ala}$ mutant appeared to be effective, at least as evidenced by abrogation of p21WAF1 induction and G1/S arrest in response to DNA damage. However, to provide a complementary, and potentially more reliable, method of p53 inactivation, we also employed HPVE6 expression, which promotes ubiquitination and degradation of p53 protein (Scheffner et al, 1990). Some indication of a more complete loss of p53 function was indeed seen, in that E6 not only blocked the DNA damage response, but also eliminated the low, basal level of p21 expression seen in untreated cells. As with mp53, however, no evidence for progression of the transformed phenotype by E6 could be observed.

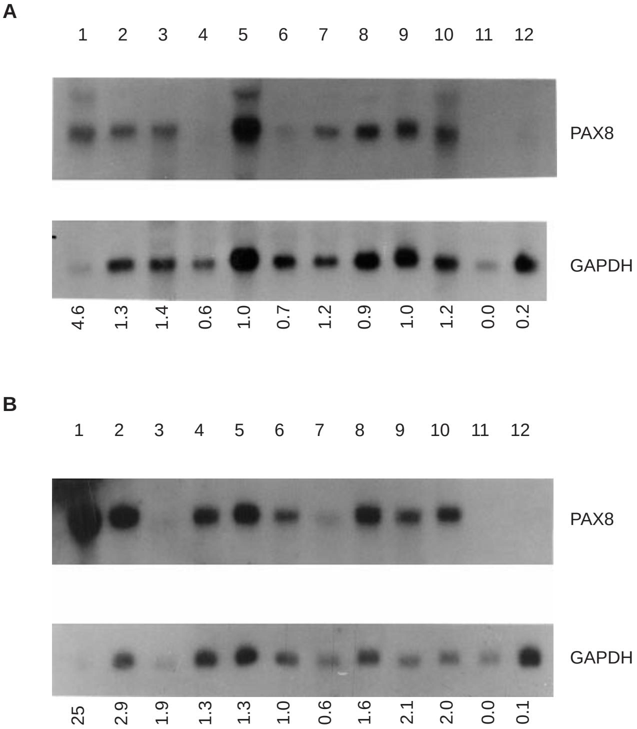

**Figure 6** Abrogation of p53 function fails to cause loss of differentiation in K1 clones, as determined by retention of PAX8 expression. Northern blot analyses of PAX8 mRNA (upper panels; transcript size 3.1 kb) compared to GAPDH (lower panels), with ratio of signals shown below each lane. (**A**) Clones K1neo 3, 5 and 11 (lanes 3–5) show similar PAX8/GAPDH ratios to mp53-expressing clones K1scx 3, 6 and 9 (lanes 6–8), as do the K1 parent line (lane 2) and two similar thyroid cancer cell lines with retained wt p53 function (K2 and K5) (lanes 9, 10). In contrast PAX8 was undetectable in a poorly-differentiated thyroid cancer line FTC133 (lane 12). (**B**) Corresponding analysis for five K1E6 clones (lanes 2–6) and their four K1neo control clones (lanes 7–10). For both blots, RNA from non-neoplastic thyroid tissue (Graves' goitre) was included as a positive control (lane 1), and from normal diploid fibroblasts as a negative control (lane 11)

*© Cancer Research Campaign 1999* 

Other groups have attempted to reconstruct p53-dependent tumour progression in other tumour models, notably with respect to multi-stage colon carcinogenesis, in which p53 mutation appears to play a role in progression of adenomas to carcinomas. Using an approach similar to our own, Williams et al (1995) also failed, however, to demonstrate any phenotypic effect of mp53 expression in an adenoma-derived cell line. Although, with some of the mutants used, this could be explained by incomplete dominant-negative activity, this could not account for the lack of effect of the $his^{273}$ mutant. Likewise, in myeloid malignancies, although there is a clear-cut association between p53 mutation and the progression of chronic myeloid leukaemia (CML) to 'blast crisis', only minor effects could be demonstrated on introduction of the $ala^{143}$ mutant into CML-derived cells (Bi et al, 1994). Clearly, the conclusion from all of these studies is that p53 mutation, while necessary, is not *sufficient* for tumour progression.

While this conclusion is entirely consistent with the role of p53 in control of cell proliferation (Wynford-Thomas, 1996), it was perhaps less predictable in relation to the now substantial body of data supporting a role for p53 in the control of tissue-specific differentiation (Almog and Rotter, 1997).

In addition to correlative evidence based on changes in p53 protein content, or (more importantly) transactivational activity accompanying differentiation (Aloni-Grinstein et al, 1993; Halevy, 1993; Weinberg et al, 1995) there is good experimental evidence that manipulation of p53 activity can modulate the differentiation process. For example, inhibition of wt p53 by dominant-negative mutants has been shown to block differentiation in B-cell (Aloni-Grinstein et al, 1993, 1995; Halevy, 1993; Weinberg et al, 1995), myeloid (Soddu et al, 1996), and muscle (Soddu et al, 1996) cell models. Conversely, introduction of wt p53 can restore the differentiation pathway to p53-null cells of B-cell (Shaulsky et al, 1991), myeloid (Soddu et al, 1994), erythroid (Feinstein et al, 1992) or epithelial (Brenner et al, 1994) origin. Indeed, two groups have reported similar data in relation to thyroid. Fagin et al (1996) partially restored differentiation (TPO and PAX8 expression) to a poorly differentiated thyroid cancer line by expression of wt p53, and Moretti et al (1997) were successful in restoring TSH-dependent expression of thyroid differentiation genes (Tg and TPO) to the undifferentiated (anaplastic) cancer cell line ARO using a temperature-sensitive p53 mutant (Val 135). However, it should be noted that the first report was restricted to a single clone derived from a line which was not representative of truly undifferentiated thyroid cancer, and in the second study, the absolute levels of transcription achieved were extremely limited (being detectable only by reverse transcription–polymerase chain reaction).

While in some of the above examples, it is difficult to exclude an indirect effect of p53 via its action on accompanying changes in cell proliferation, there is evidence in at least some models (Soddu et al, 1996) for a specific effect on differentiation, and indeed some data to demonstrate a direct action of wt p53 on transcription of differentiation genes (Aloni-Grinstein et al, 1993).

The above data therefore suggest that p53 function is necessary for completion of the differentiation programme in many cell types. While at first sight this contradicts the apparently normal differentiation of most lineages in p53-null mice, as pointed out previously (Eizenberg et al, 1996), it is entirely possible that redundant controls exist in normal cells in vivo and that it is only when these are lost following in vitro culture and/or transformation that the role of p53 is revealed.

Even in cell line models, however, there is little evidence to show that loss of p53 function can actually *reverse* differentiation (as opposed to blocking its execution) and that it can thereby contribute directly to loss of tumour cell differentiation. Interestingly, one of the most clear-cut studies in this respect (Battista et al, 1995) was carried out using a rat thyroid epithelial cell line (PCCL3), in which expression of a dominant-negative p53 ($143^{ala}$) was shown to cause loss of expression of thyroid-specific differentiation genes (Tg, TPO, TSHR), along with the thyroid-specific transcription factor PAX8. Furthermore, forced expression of PAX8 in such cells was enough to restore differentiation.

This finding contrasts strikingly with our failure to observe any diminution of PAX8 expression in our human thyroid cell line following expression of mp53 or E6. We can only speculate that the difference in behaviour reflects differences in co-existing genetic abnormalities or in the species of origin of the two cell lines. With respect to the latter, it should be noted that, in common with nearly all human (as opposed to rodent) thyroid cell lines, K1 lack expression of Tg and TPO. This appears to reflect a spontaneous and unavoidable loss of these differentiation markers in monolayer cultures of thyrocytes. Nevertheless, they retain PAX8, which lies upstream of Tg and TPO in the differentiation pathway and represents one of the fundamental determinants of thyroid differentiation. Since this is a key distinguishing feature between differentiated and undifferentiated cancers (Fabbro et al, 1994), we consider K1 to be a useful, although clearly not ideal, model for this purpose.

The conclusions from this work are entirely consistent with our findings in a different experimental model of thyroid tumour development (Bond et al, 1996), in which we observed that expression of SV40T (which abrogates p53 function) is sufficient only to induce differentiated clones with limited proliferative potential. De-differentiated sub-clones with extended lifespan only arose after a variable period of continuous culture, again pointing to a model in which anaplastic progression requires loss of p53 function together with at least one additional genetic or epi-genetic event.

Quite apart from its direct relevance for thyroid tumour biology, the clones described here represent an ideal panel of matched, isogenic cell lines with which to investigate the role of wt p53 in physiological and pathological cellular responses, an approach whose value has recently been emphasized by Weinstein et al (1997). A major limitation of most resources in this area, including the NCI panel (O'Connor et al, 1997) is that the majority of randomly selected human cancer lines do not tolerate the presence of wt p53, hence by definition it is impossible to obtain an isogenic matched line with wt function.

The starting point therefore must be, as in this study, a tumour cell type that usually retains wt function. Furthermore, it should also be indifferent to loss of this activity in terms of its proliferative phenotype in normal culture conditions, so as to minimize the possibility of differences being merely an indirect consequence of loss of p53 function. Finally, multiple independent sister clones, rather than pools, should be examined. This avoids the pitfall that can arise if an unsuspected minor sub-population of cells in the parent line have acquired an additional abnormality that by itself confers no growth advantage but that synergizes with loss of p53 function, allowing it to become the dominant clone in a pool of transfectants.

 © Cancer Research Campaign 1999

The panel of p53 +/– sister clones described here meets all these criteria and should be a useful tool to investigate the p53-dependence of a wide variety of cellular responses to external stimuli, particularly the sensitivity to chemotherapeutic agents or other novel therapies. Although other wt p53 human cell lines have been used as the 'parent' in such approaches, notably RKO colon, MCF7 breast (Fan et al, 1995), and A2780 ovarian (McIlwrath et al, 1994), to our knowledge this thyroid-derived panel represents the most well-characterized yet available.

### ACKNOWLEDGEMENTS

We are grateful to Dr Martin Schlumberger (Villejuif, France), Dr Peter Goretzki (Dusseldorf, Germany) and Dr Denise Galloway (Seattle, WA, USA) for cell lines and retroviral vectors and to Dr Bert Vogelstein (Baltimore, MD, USA), Dr Giuseppe Damante (Udine, Italy) and Dr Christophe (Brussels, Belgium) for plasmids. Grant support was provided by the Cancer Research Campaign. We thank Theresa King for manuscript preparation.

### REFERENCES

Almog N and Rotter V (1997) Involvement of p53 in cell differentiation and development. *Biochim Biophys Acta* **133**: F1–F27

Aloni-Grinstein R, Zan-Bar I, Alboum R, Goldfinger N and Rotter V (1993) Wild-type p53 functions as a control protein in the differentiation pathway of the B-cell lineage. *Oncogene* **8**: 3297–3305

Aloni-Grinstein R, Schwartz D and Rotter V (1995) Accumulation of wild-type p53 protein upon $\gamma$ irradiation induces a $G_2$ arrest-dependent immunoglobulin $\kappa$ light chain gene expression. *EMBO J* **14**: 1392–1401

Baker SJ, Markowitz S, Fearon ER, Willson JKV and Vogelstein B (1990) Suppression of human colorectal carcinoma cell growth by wild-type p53. *Science* **249**: 912–915

Barnes DM, Dublin EA, Fisher CJ, Levison DA and Millis RR (1993) Immunohistochemical detection of p53 protein in mammary carcinoma: an important new independent indicator of prognosis? *Human Pathol* **24**: 469–476

Battista S, Martelli ML, Fedele M, Chiappetta G, Trapasso F, De Vita G, Battaglia C, Santoro M, Viglietto G, Fagin JA and Fusco A (1995) A mutated p53 gene alters thyroid cell differentiation. *Oncogene* **11**: 2029–2037

Bi S, Barton CM, Lemoine NR, Cross NCP and Goldman JM (1994) Retroviral transduction of Philadelphia-positive chronic myeloid leukaemia cells with a human mutant p53 cDNA and its effect on in vitro proliferation. *Exp Hematol* **22**: 95–99

Blaydes JP, Schlumberger M, Wynford-Thomas D and Wyllie FS (1995) Interaction between p53 and TGFb1 in control of epithelial cell proliferation. *Oncogene* **10**: 307–317

Bond JA, Dawson T, Lemoine NR and Wynford-Thomas D (1992) Effect of serum growth factors and phorbol ester on growth and survival of human thyroid epithelial cells expressing mutant ras. *Mol Carcinogen* **5**: 129–135

Bond JA, Blaydes JP, Rowson J, Haughton MF, Smith JR, Wynford-Thomas D and Wyllie FS (1995) Mutant p53 rescues human diploid cells from senescence without inhibiting the induction of SDI1/WAF1. *Cancer Res* **55**: 2404–2409

Bond JA, Ness GO, Rowson J, Ivan M, White D and Wynford-Thomas D (1996) Spontaneous de-differentiation correlates with extended lifespan in transformed thyroid epithelial cells: An epigenetic mechanism of tumour progression? *Int J Cancer* **67**: 563–572

Brash DE, Reddel RR, Quanrad M, Yang K, Farrell MP and Harris CC (1987) Strontium phosphate transfection of human cells in primary culture: stable expression of the Simian virus 40 large-T-Antigen gene in primary human bronchial epithelial cells. *Mol Cell Biol* **7**: 2031–2034

Brenner L, Munozantonia T, Vellucci VF, Zhou Z-L and Reiss M (1994) Wild-type p53 tumour suppressor gene restores differentiation of human squamous carcinoma cells but not the response to transforming growth factor-beta. *Cell Growth Diff* **4**: 993–1004

Brocas H, Christophe D, Pohl V and Vassart G (1982) Cloning of human thyroglobulin complementary DNA. *FEBS Lett* **137**: 189–192

Burns JS, Lemoine L, Lemoine NR, Williams ED and Wynford-Thomas D (1989) Thyroid epithelial cell transformation by a retroviral vector expressing SV40 large T. *Br J Cancer* **59**: 755–760

Carcangiu ML, Steeper T, Zampi G and Rosai J (1985) Anaplastic thyroid carcinoma. *Am J Cell Pathol* **83**: 135–158

Challeton C, Branea F, Schlumberger M, Gaillard N, DeVathaire F, Badie C, Antonini P and Parmentier C (1997) Characterisation and radiosensitivity at high or low dose rate of four cell lines derived from human thyroid tumours. *Int J Radiat Oncol Biol Physics* **37**: 163–169

Diller L, Kassel J, Nelson CE, Gryka MA, Litwak G, Gebhardt M, Bressac B, Ozturk M, Baker SJ, Vogelstein B and Friend SH (1990) p53 functions as a cell cycle control protein in osteosarcomas. *Mol Cell Biol* **10**: 5772–5781

Eizenberg O, Faber-Elman A, Gottlieb E, Oren M, Rotter V and Schwartz M (1996) p53 plays a regulatory role in differentiation and apoptosis of central nervous system-associated cells. *Mol Cell Biol* **16**: 5178–5185

El-Deiry WS, Harper JW, O'Connor PM, Velculescu VE, Canman CE, Jackman J, Pietenpol JA, Burrell M, Hill DE, Wang Y, Wiman KG, Mercer WE, Kastan MB, Kohn KW, Elledge SJ, Kinzler KW and Vogelstein B (1994) WAF1/CIP1 is induced in p53-mediated G1 arrest and apoptosis. *Cancer Res* **54**: 1169–1174

Fabbro D, Di Loreto C, Beltrami CA, Belfiore A, Di Lauro R and Damante G (1994) Expression of thyroid-specific transcription factors TTF-1 and PAX-8 in human thyroid neoplasms. *Cancer Res* **54**: 4744–4749

Fagin JA, Matsuo K, Karmakar A, Chen DL, Tang S-H and Koeffler HP (1993) High prevalence of mutations of the p53 gene in poorly differentiated human thyroid carcinomas. *J Clin Invest* **91**: 179–184

Fagin JA, S-H, T, Zeki K, Di Lauro R, Fusco A and Gonsky R (1996) Re-expression of thyroid peroxidase in a derivative of an undifferentiated thyroid carcinoma cell line by introduction of wild-type p53. *Cancer Res* **56**: 765–771

Fan S, Smith ML, Rivet DJ, Duba D, Zhan Q, Kohn KW, Fornace AJ and O'Connor PM (1995) Disruption of p53 function sensitises breast cancer MCF-7 cells to cisplatin and pentoxifylline. *Cancer Res* **55**: 1649–1654

Fearon ER and Vogelstein BA (1990) A genetic model for colorectal tumorigenesis. *Cell* **61**: 759–767

Feinstein E, Gale RP, Reed J and Canaani E (1992) Expression of the normal p53 gene induces differentiation of K562 cells. *Oncogene* **7**: 1853–1857

Forrester K, Lupold SE, Ott VL, Chay CH, Band V, Wang XW and Harris CC (1995) Effects of p53 mutants on wild-type p53-mediated transactivation are cell type dependent. *Oncogene* **10**: 2103–2111

Friedlander P, Haupt Y, Prives C and Oren M (1996) A mutant p53 that discriminates between p53-responsive genes cannot induce apoptosis. *Mol Cell Biol* **16**: 4961–4971

Fujimoto K, Yamada Y, Okajima E, Kakizoe T, Sasaki H, Sugimura T and Terada M (1992) Frequent association of p53 gene mutation in invasive bladder cancer. *Cancer Res* **52**: 1393–1398

Greenblatt MS, Bennett WP, Hollstein M and Harris CC (1994) Mutations in the p53 tumour suppressor gene: clues to cancer etiology and molecular pathogenesis. *Cancer Res* **54**: 4855–4878

Guazzi S, Price M, De Felice M, Damante G, Mattei M-G and Di Lauro R (1990) Thyroid nuclear factor 1 (TTF1) contains a homeodomain and displays a novel DNA binding specificity. *EMBO J* **9**: 3631–3639

Halbert CL, Demers GW and Galloway DA (1991) The E7 gene of human papillomavirus type 16 is sufficient for immortalization of human epithelial cells. *J Virol* **65**: 473–478

Halevy O (1993) p53 gene is up-regulated during skeletal muscle cell differentiation. *Biochem Biophy Res Commun* **192**: 714–719

Harlow E, Crawford LV, Pim DC and Williamson NM (1981) Monoclonal antibodies specific for simian virus 40 tumour antigens. *J Virol* **39**: 861–869

Hsu TC, Johnston DA, Cherry LM, Ramkisson D, Schantz SP, Jessup JH, Winn RJ, Shirley L and Furlong C (1989) Sensitivity to genotoxic effects of bleomycin in humans; possible relationship to environmental carcinogenesis. *Int J Cancer* **43**: 403–409

Ito T, Seyama T, Mizuno T, Tsuyama N, Hayashi T, Hayashi Y, Dohi K, Nakamura N and Akiyama M (1992) Unique association of p53 mutations with undifferentiated but not with differentiated carcinomas of the thyroid gland. *Cancer Res* **52**: 1369–1371

Johnson P, Gray D, Mowat M and Benchimol S (1991) Expression of wild-type p53 is not compatible with continued growth of p53-negative tumour cells. *Mol Cell Biol* **11**: 1–11

Kastan MB, Onyekwere O, Sidransky D, Vogelstein B and Craig RW (1991) Participation of p53 protein in the cellular response to DNA damage. *Cancer Res* **51**: 6304–6311

Kemp CJ, Donehower LA, Bradley A and Balmain A (1993) Reduction of p53 gene dosage does not increase initiation or promotion but enhances malignant progression of chemically induced skin tumours. *Cell* **74**: 813–822

Lavigeur A, Maltby V, Mock D, Rossant J, Pawson T and Bernstein A (1989) High incidence of lung, bone and lymphoid tumours in transgenic mice overexpressing mutant alleles of the p53 oncogene. *Mol Cell Biol* **9**: 3982–3991

© Cancer Research Campaign 1999

Libert F, Ruel J, Ludgate M, Swillens S, Alexander N, Vassart G and Dinsart C (1987) Complete nucleotide sequence of the human thyroperoxidase-microsomal antigen cDNA. *Nucleic Acids Res* **15**: 6735

Libert F, Lefort A, Gerard C, Parmentier M, Perret J, Ludgate M, Dumont JE and Vassart G (1989) Cloning, sequencing and expression of the human thyrotropin (TSH) receptor: evidence for binding of autoantibodies. *Biochem Biophys Res Commun* **165**: 1250–1255

Ludwig RL, Bates S and Vousden K (1996) Differential activation of target cellular promoters by p53 mutants with impaired apoptotic function. *Mol Cell Biol* **66**: 4952–4960

McIlwrath AJ, Vasey PA, Ross GM and Brown R (1994) Cell cycle arrests and radiosensitivity of human tumour cell lines: dependence on wild-type p53 for radiosensitivity. *Cancer Res* **54**: 3718–3722

Michalovitz D, Halevy O and Oren M (1991) p53 mutations: gains or losses? *J Cell Biochem* **45**: 22–29

Moretti F, Farsetti A, Soddu S, Misiti S, Crescenzi M, Filetti S, Andreoli M, Sacchi A and Pontecorvi A (1997) p53 re-expression inhibits proliferation and restores differentiation of human thyroid anaplastic carcinoma cells. *Oncogene* **14**: 729–740

Navone NM, Troncoso P, Pisters LL, Goodrow TL, Palmer JL, Nichols WW, Von Eschenbach AC and Conti CJ (1993) p53 protein accumulation and gene mutation in the progression of human prostate carcinoma. *J Natl Cancer Inst* **85**: 1657–1669

O'Connor PM, Jackman J, Bae I, Myers TG, Fan S, Mutoh M, Scudiero DA, Monks A, Sausville EA, Weinstein JN, Friend S, Fornace AJ and Kohn KW (1997) Characterisation of the p53 tumor suppressor pathway in cell lines of the National Cancer Institute anticancer drug screen and correlations with the growth-inhibitory potency of 123 anticancer agents. *Cancer Res* **57**: 4285–4300

Poleev A, Fickenscher H, Mundlos S, Winterpacht A, Zabel B, Fidler A, Gruss P and Plachov D (1992) PAX8, a human paired box gene: isolation and expression in developing thyroid, kidney and Wilms' tumors. *Development* **116**: 611–623

Scheffner M, Werness BA, Huibregtse JM, Levine AJ and Howley PM (1990) The E6 oncoprotein encoded by human papillomavirus types 16 and 18 promotes the degradation of p53. *Cell* **63**: 1129–1136

Shaulsky G, Goldfinger N, Peled A and Rotter V (1991) Involvement of wild-type p53 in pre-B-cell differentiation in vitro. *Proc Natl Acad Sci USA* **88**: 8982–8986

Soddu S, Blandino G, Citro G, Scardigli R, Piaggio G, Ferber A, Calabretta B and Sacchi A (1994) Wild-type p53 gene expression induces granulocytic differentiation of HL-60 cells. *Blood* **83**: 2230–2237

Soddu S, Blandino G, Scardigli R, Coen S, Marchetti A, Rizzo MG, Bossi G, Cimino L, Crescenzi M and Sacchi A (1996) Interference with p53 protein inhibits hematopoietic and muscle differentiation. *J Cell Biol* **134**: 193–204

Southern PJ and Berg P (1982) Transformation of mammalian cells to antibiotic resistance with a bacterial gene under control of the SV40 early region promoter. *J Mol App Genet* **1**: 327–341

Weinberg WC, Azzoli CG, Chapman K, Levine AJ and Yuspa SH (1995) p53-mediated transcriptional activity increases in differentiating epidermal keratinocytes in association with decreased p53 protein. *Oncogene* **10**: 2271–2279

Weinstein JN, Myers TG, O'Connor PM and Friend SH (1997) An information-intensive approach to the molecular pharmacology of cancer. *Science* **275**: 343–349

Williams AC, Miller JC, Collard TJ, Bracey TC, Cosulich S and Paraskeva C (1995) Mutant p53 is not fully dominant over endogenous wild type p53 in a colorectal adenoma cell line as demonstrated by induction of MDM2 protein and retention of a p53 dependent G1 arrest after γ irradiation. *Oncogene* **11**: 141–149

Williams DW and Williams ED (1989) The pathology of follicular thyroid epithelial tumours. In Wynford-Thomas D and Williams ED (eds), *Thyroid Tumours: Molecular Basis of Pathogenesis*, pp. 57–65, Churchill Livingstone: Edinburgh

Wright PA, Lemoine NR, Goretzki PE, Wyllie FS, Bond J, Hughes C, Roher H-D, Williams ED and Wynford-Thomas D (1991) Mutation of the p53 gene in a differentiated human thyroid carcinoma cell line, but not in primary thyroid tumours. *Oncogene* **6**: 1693–1697

Wyllie FS, Lemoine NR, Williams ED and Wynford-Thomas D (1989) Structure and expression of nuclear oncogenes in multi-stage thyroid tumorigenesis. *Br J Cancer* **60**: 561–565

Wyllie FS, Lemoine NR, Barton CM, Dawson T, Bond JA and Wynford-Thomas D (1993) Direct growth stimulation of normal human epithelial cells by mutant p53. *Mol Carcinogen* **7**: 83–88

Wyllie FS, Haughton MF, Blaydes JP, Schlumberger M and Wynford-Thomas D (1995) Evasion of p53-mediated growth control occurs by three alternative mechanisms in transformed thyroid epithelial cells. *Oncogene* **10**: 49–59

Wynford-Thomas D (1993) Molecular genetics of thyroid cancer. *Trends Endocrinol Metab* **4**: 224–232

Wynford-Thomas D (1996) p53: guardian of cellular senescence. *J Pathol* **180**: 118–121

Wynford-Thomas D, Bond JA, Wyllie FS, Burns J, Williams ED, Jones T, Sheer D and Lemoine NR (1990) Conditional immortalisation of human thyroid epithelial cells: a tool for analysis of oncogene action. *Mol Cell Biol* **10**: 5365–5377

Wynford-Thomas D, Jones CJ and Wyllie FS (1996) The tumour suppressor gene p53 as a regulator of proliferative life-span and tumour progression. *Biol Signals* **5**: 139–153

Ziegler A, Jonason AS, Leffell DJ, Simon JA, Sharma HW, Kimmelman J, Remington L, Jacks T and Brash DE (1994) Sunburn and p53 in the onset of skin cancer. *Nature* **372**: 773–777

*© Cancer Research Campaign 1999*
